# The role of inflammation on coronary microvascular dysfunction and potential therapeutic targets

**DOI:** 10.1093/ehjopen/oeag153

**Published:** 2026-09-17

**Authors:** Wanqi Wang, Martina Magistri, John Beltrame, Noel Bairey Merz, Roxana Mehran, Vijay Kunadian

**Affiliations:** Translational and Clinical Research Institute, Faculty of Medical Sciences, Newcastle University, 4th Floor William Leech Building, Newcastle upon Tyne NE2 4HH, UK; Translational and Clinical Research Institute, Faculty of Medical Sciences, Newcastle University, 4th Floor William Leech Building, Newcastle upon Tyne NE2 4HH, UK; Division of Cardiology, Department of Medicine, University of Verona, Verona 37129, Italy; The University of Adelaide, Adelaide 5005, Australia; Cedars-Sinai Medical Center, CA 90048, USA; Mount Sinai Medical Centre, New York, NY 10029, USA; Translational and Clinical Research Institute, Faculty of Medical Sciences, Newcastle University, 4th Floor William Leech Building, Newcastle upon Tyne NE2 4HH, UK; Cardiothoracic Centre, Freeman Hospital, Newcastle upon Tyne Hospitals NHS Foundation Trust, Newcastle upon Tyne NE7 7DN, UK

**Keywords:** Inflammation, Coronary microvascular dysfunction, Pathophysiology, Therapy

## Abstract

Coronary microvascular dysfunction (CMD) is a major underlying cause of myocardial ischaemia and is associated with adverse cardiovascular outcomes. It occurs both in the presence of obstructive coronary artery disease (CAD) and in patients with non-obstructive CAD. Emerging evidence highlights chronic inflammation as a key factor in CMD pathogenesis. This review conceptualizes inflammation as a driver, amplifier, or consequence of CMD, depending on the underlying clinical phenotype. Pro-inflammatory pathways impair endothelial function and enhance immune cell infiltration within the coronary microcirculation, leading to myocardial ischaemia. Moreover, several chronic inflammatory conditions such as diabetes mellitus, obesity, and autoimmune diseases are linked with CMD. These conditions promote elevated circulating levels of pro-inflammatory cytokines, including high-sensitivity C-reactive protein, interleukins, tumour necrosis factor-α, and markers of monocyte activation. Inflammatory mechanisms of tissue damage—including adipose tissue inflammation, endothelial glycocalyx degradation, pericyte dysfunction, microvascular-specific immune cell infiltration, inflammasome signalling, microRNA dysregulation, and shear stress-induced inflammation—have been implicated in CMD. Recent advances in molecular and imaging biomarkers provide new opportunities for detecting and characterizing inflammation-driven CMD, although inflammation-specific diagnostic tools remain limited. While several therapies with anti-inflammatory or pleiotropic effects, including statins and sodium-glucose cotransporter-2 inhibitors, have demonstrated cardiovascular benefit, robust trials specifically evaluating coronary microvascular endpoints are scarce. This narrative review focuses on the chronic inflammatory mechanisms contributing to CMD, the available diagnostic techniques, and potential therapeutic strategies aimed at modulating inflammation to improve coronary microvascular function and cardiovascular outcomes.

## Graphical Abstract

**Graphical Abstract:**
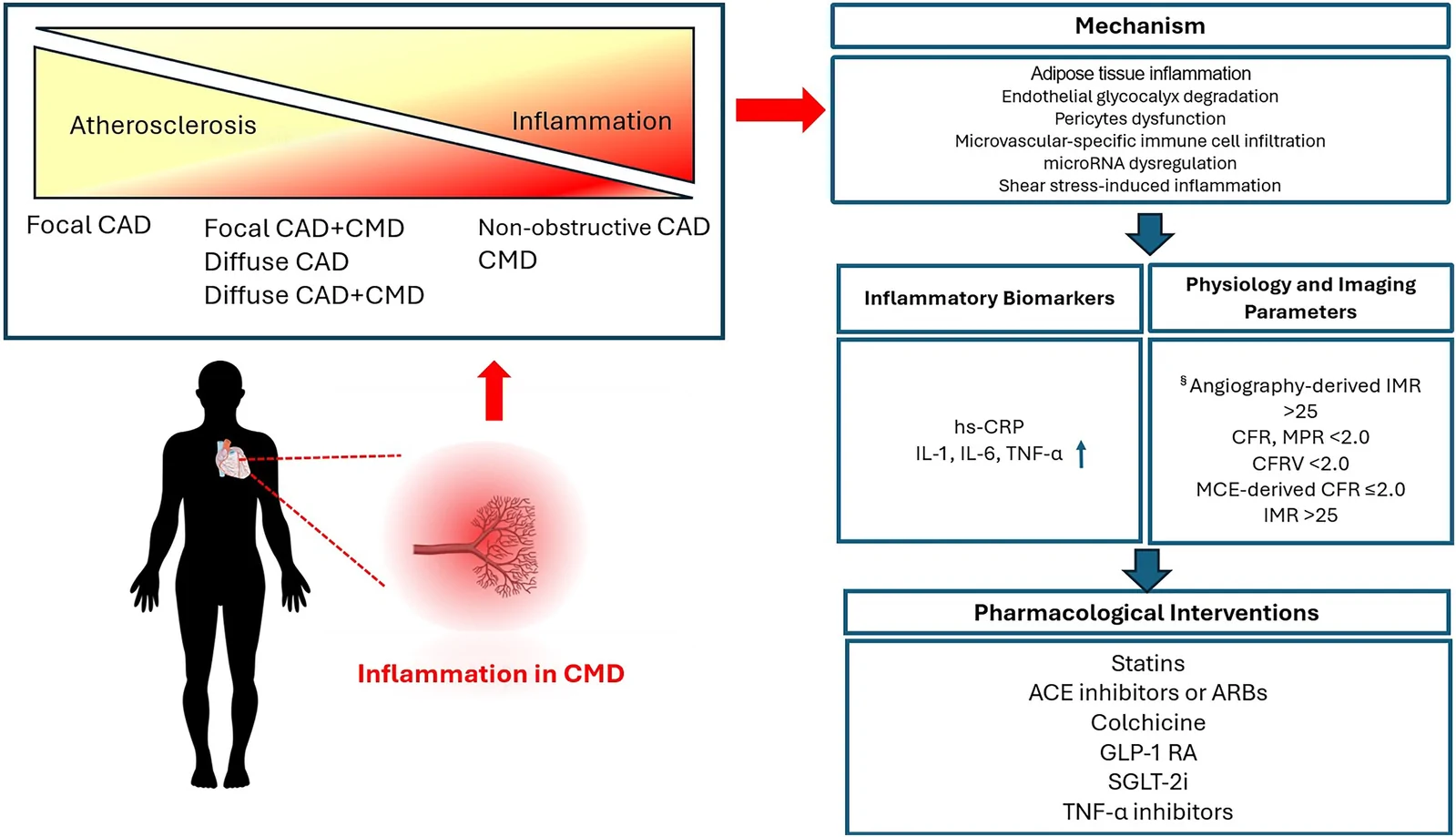

## Introduction

Coronary microvascular dysfunction (CMD) is increasingly recognized as an important contributor to ischaemic heart disease (IHD), extending beyond the traditional focus on obstructive coronary artery disease (CAD).^1^ CMD involves endothelial dysfunction, impaired vasodilation, enhanced vasoconstriction, and microvascular remodelling, which collectively lead to inadequate myocardial perfusion in the absence of major epicardial coronary artery obstruction. Over the past few decades, clinical studies have predominantly centred on atherosclerosis of the epicardial arteries. However, accumulating evidence indicates that CMD significantly influences the prognosis of patients both with and without CAD. Although it is difficult to detect by conventional imaging, CMD challenges the traditional stenosis-centred pathophysiology of IHD and reveals a critical gap in current diagnostic and therapeutic strategies. CMD has been increasingly recognized across a spectrum of clinical conditions, including myocardial ischaemia with non-obstructive coronary arteries (INOCA) and myocardial infarction with non-obstructive coronary arteries (MINOCA),^2–4^ heart failure with preserved ejection fraction (HFpEF), and Takotsubo syndrome. In addition, it is frequently associated with metabolic and inflammatory states such as metabolic syndrome,^4–6^ rheumatoid arthritis,^7,8^ post-COVID syndrome,^9^ and postmenopausal state^10,11^ (*Figure 1*).

**Figure 1:**
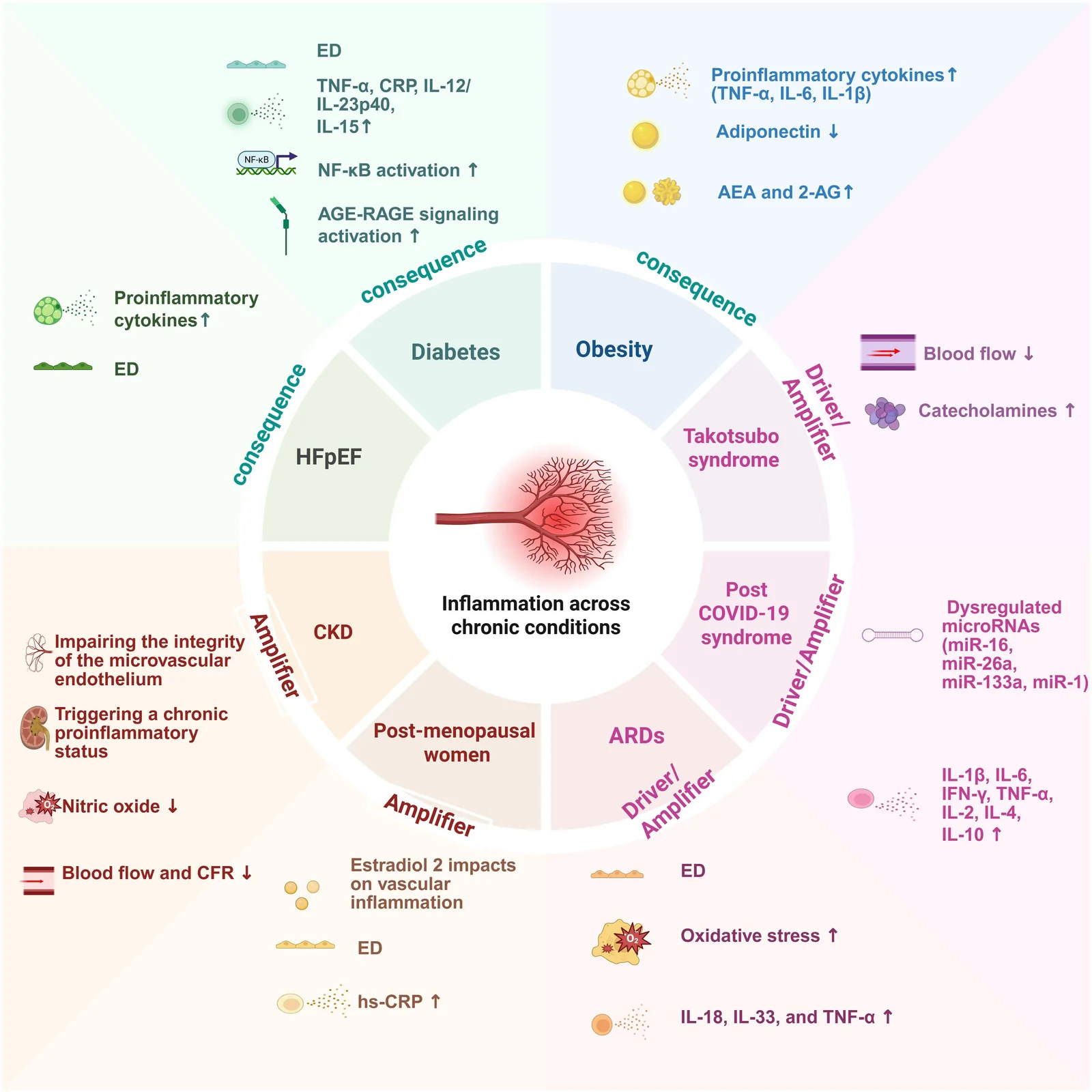
Summary of the CMD in related clinical conditions and potential mechanisms closely related to inflammation. 2-AG, 2-arachidonoylglycerol; AEA, anandamide; AGE-RAGE, advanced glycation end-products–receptor for advanced glycation end-products; ARDs, autoimmune rheumatic diseases; CKD, chronic kidney disease; CMD, coronary microvascular dysfunction; CFR, coronary flow reserve; COVID-19, coronavirus disease 2019; CRP, C-reactive protein; EAT, epicardial adipose tissue; ED, endothelial dysfunction; HFpEF, heart failure with preserved ejection fraction; hs-CRP, high-sensitivity C-reactive protein; IFN-γ, interferon gamma; IL-1β, interleukin 1 beta; IL-2, interleukin 2; IL-4, interleukin 4; IL-6, interleukin 6; IL-10, interleukin 10; IL-12/IL-23p40, interleukin 12/interleukin 23 p40 subunit; IL-15, interleukin 15; IL-18, interleukin 18; IL-33, interleukin 33; MDC, macrophage-derived chemokine; miR-1, MicroRNA 1; miR16, microRNA 16; miR26a, microRNA 26a; miR133a, microRNA 133a; NF-κB, nuclear factor kappa-light-chain-enhancer of activated B cells; TNF-α, tumour necrosis factor-α.

Inflammation, a fundamental immune system response, could be acute or chronic. Chronic inflammation may play a central role in CMD pathophysiology. Key features of chronic inflammation include the accumulation of inflammatory cells, particularly macrophages, lymphocytes, and plasma cells, within affected tissues. These cells release cytokines, growth factors, and enzymes that promote ongoing tissue injury and activate secondary repair responses, such as fibrosis.^12^ Chronic inflammatory status contributes to endothelial dysfunction and adipose tissue inflammation as well, which affects both epicardial arteries and microvasculature.^12^ Concurrently, mechanisms such as endothelial glycocalyx (EG) degradation,^13^ pericyte dysfunction,^14^ and shear stress-induced inflammation^15^ have a more specific impact on microcirculation, although current evidence remains preliminary. As our understanding of CMD evolves, targeting inflammation emerges as a potential therapeutic target within a precision-medicine framework. However, inflammation is unlikely to play an identical role across all CMD phenotypes. Depending on the clinical context, inflammatory activation may function as a primary driver of microvascular injury, as observed in post-myocardial infarction (MI) microvascular obstruction (MVO) and no-reflow, predominantly involving neutrophils and classically activated (M1) macrophages characteristic of acute high-grade inflammation^16^; as an amplifier of functional dysregulation in vasomotor disorders and microvascular spasm;^17^ or as a downstream consequence of cardiometabolic stress and structural remodelling in conditions such as HFpEF and diabetes-associated CMD, involving monocytes, and activated T-cells.^18^ This distinction has important practical implications, influencing which patients are most likely to benefit from anti-inflammatory or pleiotropic therapies, and which endpoints should be prioritized in future CMD-focused trials. Accordingly, we synthesize current evidence through an inflammation-centred framework, with emphasis on mechanistic plausibility and the strength of CMD-specific clinical data when evaluating diagnostic and therapeutic opportunities. Importantly, both inflammation and CMD represent biologically heterogeneous constructs rather than single pathological entities. Inflammation in the context of CMD encompasses diverse processes including circulating inflammatory biomarkers (e.g. hs-CRP and cytokines), immune-cell infiltration, adipose tissue inflammation. These markers may reflect distinct inflammatory pathways and are not necessarily interchangeable. Similarly, CMD comprises multiple functional and structural abnormalities of the coronary microcirculation. Different diagnostic modalities evaluate different physiological domains, including impaired vasodilatory reserve, increased microvascular resistance, endothelial dysfunction, and structural microvascular injury such as microvascular obstruction. Accordingly, interpretation of inflammation and CMD associations requires careful consideration of both phenotype heterogeneity and the specific biological and physiological processes being assessed (*Table 1*).

**Table 1:** Phenotype-oriented framework of inflammation in CMD

Conditions	CMD phenotype	Pathophysiology	Potential dominant inflammatory mechanism	Diagnostic testing strategy	Strength of CMD-specific evidence
INOCA/MINOCA^19–21^	CMD with non-obstructive atherosclerosis	Functional and structural microvascular dysfunction	Pro-inflammatory TNF-α–IL-6–CRP pathway	PET/CMR/TTDE assessment; Coronary angiography with CFR/IMR	^c^
Post-MI microvascular obstruction^22^	CMD with obstructive atherosclerosis	Ischaemia–reperfusion injury	Acute high-grade inflammation: activation of cell adhesion molecules and increased inflammatory cytokines such as TNF-α, IL-6, and IL-8	Coronary angiography with CFR/IMR; PET/CMR/TTDE assessment	^b^
HFpEF^23,24^	CMD with non-obstructive atherosclerosis/CMD with obstructive atherosclerosis	Microvascular rarefaction/remodelling	The systemic inflammatory state (IL-6↑, TNF-α↑), and endothelial inflammation (VCAM↑ and E-selectin↑)	PET/CMR/TTDE assessment	^b^
Diabetes^25^	CMD with non-obstructive atherosclerosis/CMD with obstructive atherosclerosis	Cardiometabolic dysfunction	Chronic low-grade inflammation, advanced glycation end-products (AGEs)	Coronary angiography with CFR/IMR; PET/CMR/TTDE assessment	^b^
Obesity	CMD with non-obstructive atherosclerosis/CMD with obstructive atherosclerosis	Cardiometabolic dysfunction	Adipose tissue inflammation with IL-1β, IL-6/TNF-α activation^26^	Coronary angiography with CFR/IMR; PET/CMR/TTDE assessment	^a^
Autoimmune rheumatic diseases^27^ (ARDs)	CMD without atherosclerosis	Systemic inflammatory disorders	Chronic systemic inflammation, increased levels of the pro-inflammatory cytokines (TNF-α and IL-6)	PET/CMR/TTDE assessment	^b^
Post-COVID syndromes^28,29^	CMD without atherosclerosis	Systemic inflammatory disorders	Endotheliitis, persistent cytokine activation	PET/CMR/TTDE assessment	^a^
Takotsubo syndrome^30,31^	CMD without atherosclerosis	Oxidative stress	Catecholamine excess with secondary inflammatory activation and endothelial dysfunction	Coronary angiography with CFR/IMR; PET/CMR/TTDE assessment	^b^
CKD	CMD with non-obstructive atherosclerosis/CMD with obstructive atherosclerosis	Structural remodelling/systemic inflammatory disorders	Chronic low-grade inflammation, oxidative stress, and endothelial dysfunction	PET/CMR/TTDE assessment	^a^

AGEs, advanced glycation end-products; ARDs, autoimmune rheumatic diseases; CFR, coronary flow reserve; CKD, chronic kidney disease; CMD, coronary microvascular dysfunction; CMR, cardiac magnetic resonance; CRP, C-reactive protein; E-selectin, endothelial selectin; HFpEF, heart failure with preserved ejection fraction; IL, interleukin; IMR, index of microcirculatory resistance; INOCA, ischaemia with non-obstructive coronary arteries; MINOCA, myocardial infarction with non-obstructive coronary arteries; PET, positron emission tomography; TNF-α, tumour necrosis factor-α; TTDE, transthoracic Doppler echocardiography; VCAM, vascular cell adhesion molecule.

^a^Limited mechanistic or animal studies evidence.

^b^Indirect evidence.

^c^CMD-specific physiological evidence.

## Literature search strategy

This narrative review was informed by a structured literature search of PubMed/MEDLINE for studies published between January 2000 and January 2026. Search terms included combinations of ‘coronary microvascular dysfunction’, ‘INOCA’, ‘microvascular angina’, ‘microvascular obstruction’, ‘inflammation’, ‘NLRP3 inflammasome’, ‘endothelial dysfunction’, ‘pericytes’, ‘glycocalyx’, ‘microRNA’, ‘SGLT2 inhibitors’, ‘GLP-1 receptor agonists’, and ‘anti-inflammatory therapy’. Reference lists of relevant articles and recent consensus statements were screened to ensure completeness. Priority was given to randomized controlled trials, prospective clinical studies, and CMD-specific physiological investigations when discussing diagnostic and therapeutic implications, whereas mechanistic and preclinical studies were included to contextualize pathophysiological pathways. Evidence was interpreted according to the degree of CMD specificity. Studies directly assessing coronary microvascular physiology or imaging-defined CMD endpoints were considered CMD-specific evidence, whereas findings derived from broader cardiovascular or inflammatory populations without direct CMD assessment were interpreted as indirect or extrapolated evidence.

## Pathophysiology of coronary microvascular dysfunction and the role of inflammation

The coronary microvasculature includes vessels smaller than 500 μm in diameter, lined by a single layer of endothelial cells and surrounded by thin walls, which makes them particularly vulnerable to inflammatory factors. In epicardial arteries, inflammatory mechanisms may originate from either the luminal or the adventitial side, depending on the trigger, with both pathways interacting dynamically in chronic vascular inflammation.^32^ In the coronary microcirculation, inflammation acts through multiple mechanisms that ultimately lead to CMD. Endothelial dysfunction reduces endothelium-derived relaxing factors (EDRFs) bioavailability and enhances inflammatory cytokine signalling, therefore decreasing coronary microvascular density and promoting rarefaction and myocardial fibrosis.^33,34^ In addition, disturbed shear stress caused by chronic inflammatory diseases may change the permeability of microvasculature, representing another potential mechanism.^35^ Furthermore, perivascular adipose tissue (PVAT) is gaining increasing attention for its role on vascular inflammation through the active production of a variety of cytokines, linking CMD and obesity.^36^ Overall, these pathological processes may occur simultaneously.

Along with CMD, vasomotor abnormalities leading to epicardial or microvascular coronary vasospasm are observed in ∼50% of patients with stable angina and non-obstructive CAD.^37^ Vasospastic angina is mainly mediated by vascular smooth muscle cells (VSMCs), which communicate with both the adventitia and its adjacent PVAT. Current evidence shows that PVAT volume was significantly increased at the spastic segment in patients with vasospastic angina when compared to controls.^38^ Moreover, CMD is frequently detected in patients with diffuse, non-obstructive coronary atherosclerosis and significant plaque burden on intravascular ultrasound. In this context, the high prevalence of risk factors and increased oxidative stress may explain the shared pathogenesis.^39^

In summary, different degrees of atherosclerosis and inflammation contribute to a spectrum of cardiovascular diseases, presenting different physiological, angiographic and diagnostic characteristics (*Figure 2*). CMD could arise from a spectrum of non-inflammatory mechanisms involving structural, functional, and extravascular compression abnormalities of the coronary microcirculation, such as arteriolar wall thickening, microvascular smooth muscle dysfunction, and myocardial hypertrophy.^40^ This heterogeneity may explain why patients with acute coronary syndrome (ACS) and concomitant CMD often experience poorer clinical outcomes. Traditional treatments—whether pharmacological or interventional—primarily target large-vessel atherosclerotic plaques, potentially leaving the underlying microvascular pathology insufficiently addressed. This review intentionally centres on pathophysiological mechanisms of CMD that intersect with inflammatory processes.

**Figure 2:**
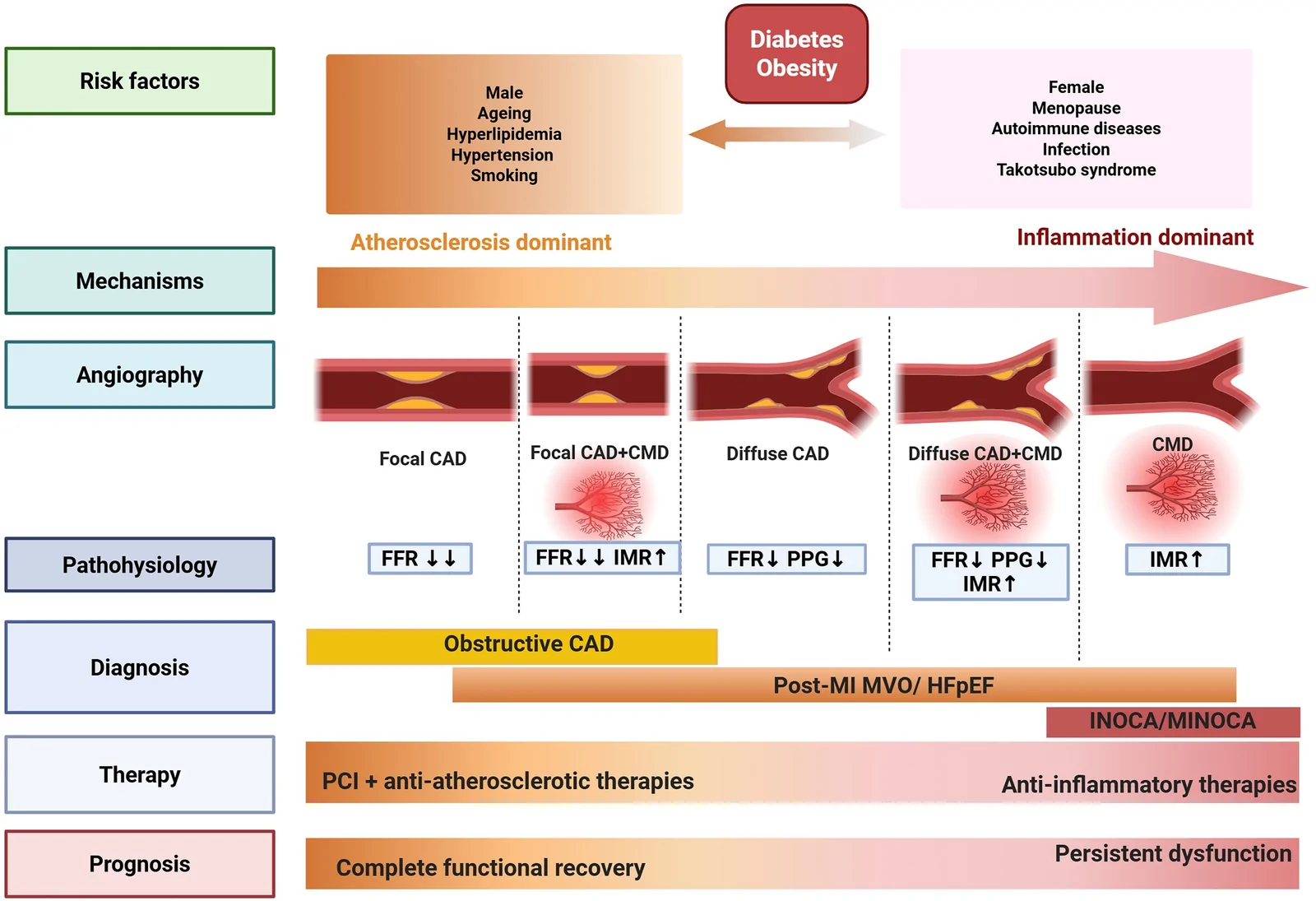
Summary of how different degrees of atherosclerosis and inflammation may contribute to a spectrum of cardiovascular diseases. CAD, coronary artery disease; CMD, coronary microvascular dysfunction; FFR, fractional flow reserve; HFpEF, heart failure with preserved ejection fraction; IMR, index of microcirculatory resistance; INOCA, ischaemia with non-obstructive coronary arteries; MI, myocardial infarction; MINOCA, myocardial infarction with non-obstructive coronary arteries; MVO, microvascular obstruction; PPG, pullback pressure gradient.

## Endothelial dysfunction and inflammation

Endothelial dysfunction is a fundamental mechanism underlying both CAD and CMD, particularly in the context of chronic inflammation and oxidative stress.^41–44^ Clinical evidence suggests that coronary microvascular endothelial dysfunction is associated with early-stage epicardial coronary atherosclerosis, as detected by virtual-histology intravascular ultrasound, in patients presenting with chest pain and non-obstructive CAD.^45^ EDRFs, including vasodilator prostaglandins, nitric oxide (NO), and endothelium-dependent hyperpolarization (EDH) factors, facilitate relaxation of VSMCs and subsequent vasodilatation.^46–48^ Under physiological conditions, NO is a primary mediator of endothelium-dependent vasodilation, especially in the epicardial coronary artery. As the blood vessel size decreases, the NO contribution to vasodilation diminishes, while EDH increases correspondingly. Consequently, EDH-mediated responses become the predominant mechanism of vasodilation in small resistance arteries.^49–51^

Inflammation reduces the bioavailability of both NO and EDRFs, thereby leading to endothelial dysfunction and promoting vasoconstriction, thrombosis, and plaque formation. These abnormalities represent an initial step in the development of coronary atherosclerosis.^52^ Moreover, when exposed to chronic inflammatory stimuli, endothelial cells undergo functional impairments that contribute to impaired myocardial perfusion.^53,54^ Inflammation up-regulates adhesion molecules on the endothelial surface, promoting leukocyte adhesion and transmigration. Pro-inflammatory cytokines, including TNF-α and interleukin (IL)-1β, activate the nuclear factor-kappa B (NF-κB) pathway, increase the expression of intercellular adhesion molecule 1 (ICAM-1), vascular cell adhesion molecule 1 (VCAM-1), and E-selectin.^55–58^ Endothelial cells also release chemokines, such as monocyte chemoattractant protein-1 (MCP-1), attracting monocytes and T-cells.^59^ Leukocyte infiltration into the microvascular wall contributes to oxidative stress and vascular remodelling, amplifying the inflammatory response. This mechanism occurs mainly after vascular endothelial damage, especially after MI. Moreover, through pro-inflammatory cytokines and the activation of the Wnt/β-catenin pathway, chronic inflammation can induce endothelial-to-mesenchymal transition (EndMT), wherein endothelial cells lose their characteristic markers and acquire a mesenchymal phenotype, via transforming growth factor-β signalling.^60,61^ This process could indicate endothelial barrier dysfunction and increase fibrosis, contributing to microvascular remodelling and dysfunction.

Thus, endothelial dysfunction can act as a trigger of microvascular inflammation, initiating a vicious cycle within the vessel wall through cytokine-mediated endothelial injury and local immune activation. In addition, extravascular sources of inflammation, such as PVAT, also contribute to inflammatory mechanisms by releasing adipokines and cytokines that impair endothelial signalling.

## Perivascular adipose tissue inflammation

PVAT is recognized as an active participant in various physiological and pathological processes, including immune regulation.^62^ Within this tissue, lymphocyte-adipocyte interactions substantially influence immune responses. PVAT produces various substances, including both pro-inflammatory (e.g. leptin, resistin, visfatin, TNF-α, and IL-6) and anti-inflammatory factors (e.g. adiponectin). Pro-inflammatory mediators are associated with insulin resistance and endothelial dysfunction, both of which increase the risk of cardiovascular disease observed in obesity and CMD.^36,63^ Epicardial adipose tissue (EAT) is a subtype of PVAT located between the myocardium and the pericardium, surrounding the coronary arteries, and contributing to CAD and CMD through inflammatory mechanisms.^64,65^ Under physiological conditions, EAT secretes protective adipokines; however, when metabolically dysregulated, it produces pro-inflammatory and pro-atherogenic adipokines that adversely affect adjacent vessels.^66^ A novel diagnostic technique using coronary computed tomography angiography (CCTA) has been developed to detect PVAT and quantify coronary inflammation. The fat attenuation index (FAI) is a novel imaging biomarker that predicts prognosis in patients with non-obstructive CAD and may serve as a potential indicator of inflammation in individuals with cardiovascular risk factors.^67,68^

In summary, adipose tissue amplifies the inflammatory cascade by releasing cytokines and adipokines that impair microvascular function, linking metabolic dysregulation to inflammation in CMD.

## Endothelial glycocalyx degradation

The EG is a thin, gel-like layer lining the luminal surface of microvessels consisting of proteoglycans, glycoproteins, and glycosaminoglycans (GAG), such as dermatan sulfate, keratan sulfate, heparan sulfate, and hyaluronic acid. Beyond serving as a physical barrier that protects the vascular endothelium, EG has a critical role in regulating cellular transduction pathways involved in proliferation, differentiation and apoptosis.^13^ It also facilitates inter-endothelial cell signalling and maintains cell–cell adhesion, thereby influencing vascular permeability and modulating wall shear stress.^69^ For these reasons, the microvasculature is highly dependent on the glycocalyx, and its degradation is especially relevant in CMD.^70^ Pro-inflammatory cytokines (e.g. TNF-α, IL-1β, IL-6) activate enzymes such as heparanase and matrix metalloproteinases, which degrade the EG, thereby increasing endothelial permeability, promoting leukocyte adhesion, and impairing NO-mediated vasodilation.^71^ Elevated serum levels of syndecan-1, one of the main components of EG, are independently linked to CMD and impaired microvascular vasodilatory capacity.^72^ This finding may offer a new avenue to identify and treat patients with CMD.

In summary, glycocalyx degradation represents a pivotal interface between inflammation and endothelial dysfunction, amplifying vascular permeability and leukocyte recruitment, and thereby perpetuating the inflammatory cascade that underlies CMD.

## Pericyte dysfunction

Pericytes have been investigated for decades in neuroscience, particularly regarding their role in blood–brain barrier function. However, their significance in coronary microcirculation and cardiovascular diseases has recently garnered attention.^73^ Pericytes are abundant mural cells located along the abluminal surface of endothelial cells within the microvasculature, whereas they are absent in larger epicardial arteries. Loss of pericytes increases microvascular permeability and disrupts the regulation of blood flow.^74^ Pericyte damage and subsequent apoptosis induced by pro-inflammatory cytokines and oxidative stress increase endothelial sensitivity to inflammatory signals, promoting fibrosis and microvascular dysfunction.^14,75^ However, pericytes can also exert anti-inflammatory effects by down-regulating IL-6 expression in endothelial cells and reducing IL-6 mRNA levels in cardiomyocytes.^76^

## Coronary microembolization and microvascular obstruction after myocardial infarction

Coronary microembolization represents an important mechanism linking inflammation with structural coronary microvascular dysfunction, particularly in the setting of acute MI.^77^ Distal embolization of thrombotic or atheromatous debris during plaque rupture or PCI can occlude arterioles and capillaries, initiating local inflammatory activation within the microcirculation.^77,78^

Clinical evidence demonstrates that prolonged total ischaemia time in ST-segment elevation myocardial infarction (STEMI) patients undergoing PCI is associated with a higher incidence of MVO on cardiac magnetic resonance (CMR) imaging.^79^ The presence of MVO correlates with larger infarct size, impaired left ventricular functional recovery, and adverse ventricular remodelling following reperfusion.^80^ Notably, MVO may occur despite successful epicardial revascularization and independently predicts worse clinical outcomes.

Emerging data from immunohistochemical studies further suggest that intracoronary thrombus composition influences the development and severity of MVO, reinforcing a mechanistic link between local pro-inflammatory mediators and downstream microvascular injury.^81^ In this context, endothelial disruption and persistent inflammatory signalling promote neutrophil and monocyte recruitment, oxidative stress, capillary plugging, and pericyte-mediated microvascular constriction, ultimately leading to sustained impairment of coronary flow reserve (CFR) and progressive ventricular remodelling.^77,82^ Recognition of microembolization and MVO as inflammation-driven processes highlights the importance of early, phenotype-specific strategies targeting inflammatory cascades in ACS.

## Microvascular-specific immune cell infiltration

A previous study using multiplex immunohistochemistry identified immune cell infiltration at various stages of CAD.^83^ T-cells, B-cells, non-M2 macrophages, and neutrophils were increased in the intima of diseased coronary arteries, along with a higher ratio of non-cytotoxic to cytotoxic T-cells compared with normal vessels.^83^

Direct interactions between endothelial and immune cells can lead to endothelial dysfunction, which is also recognized as one of the key mechanisms of CMD.^84^ The recruitment of M2 macrophages and regulatory T-cells contributes to tissue repair and immune resolution rather than acute inflammatory responses.^85^ Unlike CAD, where plaque rupture induces high-grade inflammatory activation, CMD inflammation typically manifests as systemic and persistent, driven by metabolic dysfunction and oxidative stress. Additionally, the microvascular environment—characterized by unique shear stress patterns, oxygen gradients, and endothelial signalling—facilitates specialized immune responses that differ from those in larger arteries, potentially promoting immune tolerance and vascular remodelling rather than plaque-driven inflammation.^85^

## Inflammasome

Nucleotide-binding and oligomerization domain (NOD)-like receptors (NLRs) are a recently identified family of intracellular sensors that detect microbial infections and danger signals.^86^ Innate immune responses by inducing signalling pathways, such as NF-κB, mitogen-activated protein kinases, and the caspase 1 inflammasome can be triggered by NLRs. This leads to the consequence of the activation of cytokines and chemokines.^87^ NOD-like receptor family pyrin domain-containing 3 (NLRP3) has been shown to recognize a diverse range of pathogen-associated molecular patterns (PAMPs) and various damage-associated molecular patterns (DAMPs). It has been proposed that this shared activation mechanism may involve reactive oxygen species (ROS) production or lysosomal damage.^88^ NLRP3 is a key driver of inflammation in CAD.^86,89^ It senses danger signals (e.g. ROS, cholesterol crystals) and activates caspase-1, producing IL-1β, IL-18, and IL-33. NLRP3 activation in endothelial cells and macrophages amplifies local inflammation.^90–93^ A study suggested that NLRP3 can potentially identify very high-risk (10-year risk SCORE ≥10%) and low-risk (10-year risk SCORE <1%) patients, which means NLRP3, along with other biomarkers, may better predict CAD and major adverse cardiovascular events (MACE).^94^ However, clinical evidence demonstrating that inflammasome targeting improves CMD haemodynamics remains scarce.

## MicroRNA dysregulation

MicroRNAs (miRNAs) are small non-coding RNAs responsible for post-translational gene expression, which can regulate endothelial function, angiogenesis, and inflammation within the microvasculature.^95^ Previous studies have shown that miRNAs contribute to atherosclerosis in epicardial CAD.^96^ More recently, their impact has also been investigated in the microvascular environment. For example, miR-16-2-3p has been shown to regulate fatty acid degradation in endothelial cells and to improve coronary microvascular reperfusion when overexpressed in diabetic patients with CMD.^97^ Experimental studies on animals have demonstrated that miR-126 is highly expressed in endothelial cells and plays a fundamental role in maintaining vascular integrity and promoting angiogenesis. Moreover, it regulates endothelial expression of VCAM-1, thereby modulating leukocyte adhesion and vascular inflammation.^98^

Inflammatory stimuli can alter miRNAs expression, leading to endothelial dysfunction and impaired vasodilation.^97,99^ Given the microvasculature's reliance on finely tuned endothelial function, dysregulation of these miRNAs has a more immediate and profound impact on microvascular health compared with epicardial vessels. As emerging biomarkers, miRNAs may facilitate more refined diagnostic stratification and targeted therapeutic strategies.

## Shear stress-induced inflammation

Shear stress plays a role in regulating endothelial function in the coronary microcirculation by consistently changing blood flow. Under physiological conditions, endothelial shear stress promotes anti-inflammatory Krüppel-like factor 2 signalling and endothelial NO synthase (eNOS) pathways.^100^ However, low or oscillatory shear stress, characteristic of CMD, activates endothelial mechanosensors, such as platelet endothelial cell adhesion molecule-1 (PECAM-1) and vascular endothelial growth factor receptor-2 (VEGFR-2), leading to the generation of ROS, activation of NF-κB, and up-regulation of pro-inflammatory cytokines, including IL-6 and MCP-1, as well as adhesion molecules like VCAM-1 and ICAM-1.^101,102^ This represents one of the potential molecular mechanisms involved in the early stages of atherosclerosis, increasing endothelial permeability by impairing cytoskeleton arrangement and endothelial junctions, and facilitating inflammatory cells penetration into the intima.^103^ Additionally, altered shear stress contributes to glycocalyx degradation by down-regulating GAG of proteoglycans, thereby impairing endothelial barrier function and further promoting leukocyte adhesion and inflammation.^104^ This shear-induced inflammatory cascade may play an essential role in CMD pathophysiology, particularly in conditions like INOCA, HFpEF, and diabetes. Understanding the relationship between different blood flow patterns and vascular inflammation could provide new therapeutic strategies targeting specific mechanotransduction pathways.

## Inflammation in coronary microvascular dysfunction: driver, amplifier, or consequence?

Given the multiplicity of inflammatory mechanisms described above, an important question arises: does inflammation uniformly drive CMD across all phenotypes, or does its role vary according to clinical context? In certain settings, inflammatory activation appears to function as a primary driver of microvascular injury. For example, following acute MI, inflammatory cell recruitment, cytokine release, and endothelial disruption contribute directly to MVO and no-reflow phenomena, resulting in persistent structural impairment of the microcirculation. In other contexts, inflammation may act predominantly as an amplifier of functional dysregulation. In vasomotor disorders and microvascular spasm, endothelial dysfunction, oxidative stress, and enhanced vasoconstrictor signalling can be potentiated by low-grade inflammatory activation, even in the absence of overt structural rarefaction. Here, inflammation may exacerbate abnormal vasoreactivity rather than initiate irreversible microvascular damage. Conversely, in cardiometabolic conditions such as diabetes, obesity, and HFpEF, inflammation may represent a downstream consequence of systemic metabolic stress, adipose tissue dysfunction, and neurohormonal activation. Chronic low-grade inflammation in these states promotes endothelial dysfunction, impaired NO bioavailability, glycocalyx degradation, and microvascular remodelling, thereby perpetuating a cycle of reduced CFR and myocardial dysfunction. For example, chronic impairment of CFR may result in repeated episodes of subendocardial ischaemia, promoting myocardial fibrosis, increased extracellular matrix deposition, and progressive ventricular stiffening. In HFpEF, systemic inflammation and endothelial dysfunction are thought to drive microvascular rarefaction and impaired NO–cyclic guanosine monophosphate signalling, contributing to diastolic dysfunction and reduced exercise capacity. Over time, these processes may perpetuate adverse ventricular remodelling and worsen clinical outcomes, highlighting CMD not merely as an epiphenomenon but as a potential contributor to disease progression.

Recognizing whether inflammation acts primarily as a driver, amplifier, or consequence has important therapeutic implications. Interventions targeting upstream inflammatory signalling may be most effective in phenotypes where inflammation is causative, whereas in functional vasomotor disorders, modulation of endothelial signalling and vasoconstrictor pathways may be equally or more critical. This framework underscores the need for phenotype-specific trial design and careful interpretation of anti-inflammatory strategies in CMD.

## Haemodynamic measurement and imaging modalities for assessing coronary microvascular dysfunction and inflammation

In the clinical setting, CMD can be assessed using both invasive and non-invasive approaches. Invasive physiological assessment is performed during coronary angiography using pressure- and temperature-sensitive guidewires to quantify CFR and index of microvascular resistance (IMR), with resting and hyperaemic status. Non-invasive techniques, including positron emission tomography (PET) and CMR imaging, estimate myocardial blood flow and flow reserve by comparing perfusion at rest and during pharmacological stress. More recently, angiography-derived methods enable estimation of microvascular function from standard coronary angiograms without the need for additional instrumentation or pharmacological hyperaemia. Taken together, these approaches allow CMD to be identified across different clinical settings, balancing diagnostic accuracy, invasiveness, and availability.

## Coronary microvascular dysfunction haemodynamic assessment

The main haemodynamic hallmarks of CMD are CFR and IMR. CFR quantifies the ability of the coronary circulation to increase blood flow in response to stress or vasodilatory stimuli. It is a sensitive marker of early coronary dysfunction across different cardiac diseases, such as INOCA, HFpEF, and diabetic heart disease, and it predicts adverse outcomes, including MACE and heart failure.^105^ The relationship between CFR and inflammation has recently gained attention. Taqueti *et al.* demonstrated that impaired CFR is independently associated with increased inflammation and myocardial stiffness, suggesting that inflammation disrupts the correlation between coronary blood flow and myocardial function.^106^ When combined with inflammatory markers, CFR provides deeper insights into the underlying pathophysiology of CMD and the prognosis of patients.

The IMR is an invasive physiological measurement used to specifically assess coronary microvascular function independently of epicardial coronary arteries.^107,108^ Although the pathological range of IMR values varies across studies, a threshold of <25 units is commonly used to define normal IMR in patients with stable angina and chronic coronary syndrome.^109^ IMR >40 units indicates MVO in patients with STEMI, and it is associated with poor prognosis.^110^ In recent years, angiography-derived indices of microvascular resistance have been developed alongside the use of artificial intelligence for three-dimensional vessel reconstruction. Angiography-derived IMR has demonstrated both diagnostic accuracy and prognostic value for adverse cardiovascular events comparable to that of invasive IMR.^111^ It may serve as a valuable non-invasive alternative in patients with unstable clinical conditions or in settings with limited availability of specialized equipment in the catheterization laboratory.^112^ Although IMR does not directly measure inflammation, it is a robust marker of structural microvascular resistance, particularly sensitive to inflammatory injury. Many pro-inflammatory factors have been found to be increased in CMD patients, including VCAM-1, IL-1, IL-6, TNF-α, CD40 ligand, Rho kinase, matrix metalloproteinase-9, and growth differentiation factor-15. In particular, higher IMR values have been associated with increased homocysteine levels.^113^ However, a critical unmet need in the field is the identification of inflammatory biomarkers that consistently correlate with invasive physiological indices such as IMR and CFR at the individual patient level, as this gap currently limits endotype-driven risk stratification and the rational personalization of anti-inflammatory therapy in CMD.

## Non-invasive imaging techniques

PET enables the assessment of myocardial blood flow (MBF) at rest and during drug-induced hyperaemia. This allows for the calculation of myocardial flow reserve (MFR; PET-derived CFR), defined as the ratio of stress to rest MBF, and the detection of CMD in patients without obstructive CAD.^114^ Reduced MBF is independently associated with increased systemic inflammation and impaired myocardial strain. Vaccarino *et al.* found that reduced CFR, measured by PET, was associated with higher levels of inflammation in asymptomatic male twins. These findings support the hypothesis that inflammation may directly impair coronary microvascular function.^115^ In addition, 18F-fluorodeoxyglucose positron emission tomography (18F-FDG PET) can directly assess the inflammatory burden by detecting glucose metabolism in activated immune cells.^116^ It is widely applied in atherosclerosis, vasculitis, and systemic inflammatory diseases. Moreover, new tracers like ^68^Ga-DOTATATE and ^11^C-PK11195 have enhanced the imaging of macrophage-driven inflammation in larger vessels.^117^ However, their clinical availability is extremely limited, and their efficacy in detecting inflammatory processes specific to CMD is not well-defined.

CMR imaging enables the measurement of the myocardial perfusion reserve (MPR), defined as the ratio of MBF during stress to MBF at rest. The myocardial perfusion reserve index (MPRI) is calculated from the ratio of myocardial signal-intensity upslope during stress relative to rest. MPRI >2.0 is generally considered normal, although thresholds may vary depending on acquisition technique and population studied. MPRI between 1.5 and 2.0 indicates mild impairment or possible early CMD. MPRI <1.5 typically indicates significant microvascular dysfunction.^118^ Late gadolinium enhancement (LGE) can also reflect fibrotic remodelling secondary to inflammation-driven CMD.

CCTA is a non-invasive imaging modality used to assess the severity of coronary stenosis by providing detailed anatomic images of the coronary arteries.^119^ Although CCTA cannot directly assess microvascular function, it allows quantification of the CCTA-derived FAI, which provides valuable insights into vascular inflammation and CMD.^120^ Myocardial computed tomography perfusion (CTP) imaging has shown considerable accuracy when the results are interpreted alongside CCTA findings. This integrated approach enhances diagnostic capabilities, allowing for better assessment of myocardial blood flow and overall cardiac function.^121^ Dynamic CTP, which quantifies myocardial blood flow under stress conditions, can be used to estimate CFR (CT-derived MPR). A reduced CT-derived MPR (<2.0) is a characteristic feature of CMD.^122^

Transthoracic echocardiography and myocardial contrast echocardiography (MCE) are ultrasound-based alternatives for assessing microvascular impairment and monitoring treatment response. However, both require expertise in image acquisition and interpretation to ensure diagnostic accuracy and cannot directly assess inflammation.^19^

In summary, the assessment of CMD has rapidly developed through both invasive physiological techniques and non-invasive imaging modalities. These approaches enable clinicians to identify CMD patients across different clinical scenarios, each with its advantages and limitations (*Table 2*).

**Table 2:** Coronary haemodynamic measurements and non-invasive modalities for evaluation of CMD

Coronary pathophysiology	Modality	Parameter	Diagnostic parameters	Potential inflammatory relevance	Pros	Cons
Invasive CFR	Coronary angiography	CFR = hyperaemic blood flow/rest blood flow	Normal CFR: >2.5^47–50^ Abnormal CFR: <2.0–2.5^54–56^	No direct inflammatory assessment	Gold standard Evaluated CAD and CMD	Invasive; High costs; Requires pharmacologically induced hyperaemia
CFR-derived assessment	Cardiac PET	MFR (PET-derived CFR) = hyperaemic MBF (stress)/rest MBF	Abnormal MFR (PET-derived CFR) <2^73–76^	Potential direct assessment of vascular inflammation (using special tracers ^68^Ga-DOTATATE and ^11^C-PK11195)^78–80^	Non-invasive High spatial and temporal resolution High diagnostic and prognostic value Quantitative capability Global or regional evaluation Low radiation exposure	Limited availability (requires radiotracers and specialized equipment); High costs; Requires exclusion of obstructive CAD
CFR-derived assessment	Cardiac MRI	MPR (MRI-derived CFR) = hyperaemic MBF (stress)/rest MBF MPRI = ratio of myocardial upslope during stress vs. rest	Abnormal MPR<2 MPRI<1.5^118,123^	Potential indirect assessment through tissue characterization (T1 Mapping & Extracellular volume reflecting diffuse myocardial tissue alteration, which may indicate inflammation)	Non-invasive No radiation exposure Excellent spatial resolution Tissue characterization Simultaneous assessment of ventricular function and fibrosis	Limited availability; Advanced imaging protocols; Specialized software; Experience interpreting perfusion maps
CFR-derived assessment	CTP	CT-derived MPR = hyperaemic MBF (stress)/rest MBF	Abnormal MPR <2.0	Potential indirect assessment of coronary inflammation using CCTA-derived FAI^41–43,89,90^	Combines anatomy with function High spatial resolution Simultaneous assessment of coronary stenosis	Radiation exposure, especially with dual-phase imaging Iodinated contrast (caution in renal dysfunction); Limited availability of software for quantitative MBF; Affected by motion or arrhythmias
CFR-derived assessment	TTDE	CFVR = peak diastolic velocity (stress)/peak diastolic velocity (rest)	CFVR <2	No direct inflammatory assessment	Low cost No radiation exposure Real-time, bedside test Repeated for serial monitoring	Technically challenging acquisition; Only the LAD (not global coronary circulation); Operator-dependent Measures velocity not absolute flow
CFR-derived assessment	MCE	MFR or CFR (contrast-based)	MCE-derived CFR ≤2	No direct inflammatory assessment	Low cost No radiation exposure Real-time, bedside test Repeated for serial monitoring	Limited standardization and availability of quantitative analysis; Operator-dependent
Invasive-IMR	Pressure and temperature-sensor guidewire	IMR = Pd × Tmn Pd = distal coronary pressure during hyperaemia; Tmn = mean transit time of saline bolus during hyperaemia	IMR >25 units	No direct inflammatory assessment	Less affected by haemodynamic variables than CFR Independent of epicardial stenosis Useful in acute and chronic coronary syndromes	Requires specialized equipment and expertise; Invasive
IMR-derived assessment	3D quantitative coronary angiography reconstruction	IMR_angio_ = Pd × Tmn Pd = distal coronary pressure during hyperaemia; Tmn = mean transit time of saline bolus during hyperaemia	IMR_angio_ >25 units	N/A	No pressure wire or adenosine required Can be done retrospectively using routine angiograms	Dependent on the software platform and image acquisition quality

CAD, coronary artery disease; CCTA, coronary computed tomography angiography; CFR, coronary flow reserve; CFVR, coronary flow velocity reserve; CMD, coronary microvascular dysfunction; CT, computed tomography; CTP, computed tomography perfusion; FAI, fat attenuation index; IMR, index of microcirculatory resistance; IMRangio, angiography-derived index of microcirculatory resistance; LAD, left anterior descending artery; MBF, myocardial blood flow; MCE, myocardial contrast echocardiography; MFR, myocardial flow reserve; MPR, myocardial perfusion reserve; MPRI, myocardial perfusion reserve index; MRI, magnetic resonance imaging; Pd, distal coronary pressure; PET, positron emission tomography; Tmn, mean transit time (during hyperaemia); TTDE, transthoracic Doppler echocardiography.

## Therapeutic implications and future directions

### Conventional pharmacological interventions

#### Statins

Statins are primarily used to reduce LDL cholesterol and cardiovascular risk, while their endothelial-stabilizing and pleiotropic anti-inflammatory effects support their use as an important component of therapy relevant to CMD.^124^ Monocyte chemoattractant protein-1 (MCP-1) promotes monocyte and macrophage recruitment via the C-C motif chemokine receptor-2 (CCR2). Statins inhibit the C-C motif chemokine ligand 2 (CCL2) expression, thereby reducing leukocyte infiltration and subsequent inflammatory cascades associated with atherosclerosis and MI.^125^ This down-regulation is regarded as one of the pleiotropic effects of statins, contributing to their cardiovascular protective properties. Moreover, statins effectively shift immune responses towards anti-inflammatory pathways by inhibiting TLR4-mediated activation of inflammation is foremost.^126^ This interaction not only mitigates inflammation but also stabilizes atherosclerotic plaques. Additionally, recent experimental studies have suggested that statins might modulate inflammation by reducing mitochondrial cholesterol content in macrophages, thereby promoting a shift towards an anti-inflammatory phenotype,^127^ preventing further endothelial injury, and enhancing vessel stability. Several small observational and randomized studies indicated that patients with angina and non-obstructive CAD with or without hyperlipidaemia demonstrate improved coronary microcirculation and endothelial function with statin treatment.^124^ In the CONFIRM registry study, which included a total of 10 418 patients—5712 with normal coronary arteries and 4706 with non-obstructive CAD (1–49% diameter stenosis)—a lower mortality rate was observed among patients with non-obstructive CAD who were receiving statin therapy at baseline. This association was absent in patients without evidence of CAD.^128^ Meta-analyses have demonstrated that statins significantly improve CFR in patients without coronary stenosis, irrespective of follow-up duration.^129^ Moreover, high-intensity statin therapy appears to be particularly effective in improving coronary microvascular function in patients with STEMI, where CMD frequently coexists.^130^ The improvements in microvascular resistance following statin treatment align with the notion that these pharmacological agents address both atherogenic and inflammatory components synergistically. Currently, European guidelines recommend statin treatment for all patients with chronic coronary syndrome, including those with microvascular angina.^122^

Statins exert a multifaceted influence on cardiovascular health by improving endothelial function, modulating inflammatory pathways, and stabilizing cholesterol levels. These properties make them a potential component of therapeutic strategies for the prevention and management of CMD. However, associations between statin use and improved coronary microvascular function may be influenced by confounding by indication, concomitant risk-factor management, and differences in baseline atherosclerotic burden. Moreover, reductions in inflammation may partly reflect LDL lowering and reduced vascular lipid burden rather than a direct anti-inflammatory effect on the coronary microcirculation. Further research is needed to elucidate their precise anti-inflammatory mechanisms and to better understand the relationship between statin therapy and clinical outcomes in patients with CMD.

#### Angiotensin-converting enzyme inhibitors and angiotensin II receptor blockers

Angiotensin-converting enzyme inhibitors (ACEIs) can reduce vascular inflammation by lowering angiotensin II (Ang II)-mediated oxidative stress and improving endothelial function.^131^ By inhibiting ACE, these medications can reduce the production of Ang II, a peptide that exerts pro-inflammatory effects by stimulating cytokine production (IL-6, IL-1β, and TNF-α), chemokine, and VCAM-1.^127^ Angiotensin II receptor blockers (ARBs) specifically block the effects of Ang II at the AT1 receptor and also exhibit anti-inflammatory properties. Experimental study indicates that ARBs can suppress the activation of NF-κB and other pro-inflammatory pathways, reducing cytokine release and vascular inflammation.^132^ In addition, a meta-analysis including 10 studies on ACEIs and 9 on ARBs suggested that, among patients without obstructive CAD, treatment improved CFR after 12 months of follow-up.^129^ Furthermore, a recent systematic review and meta-analysis of randomized clinical trials demonstrated that patients with microvascular angina have improvement in CFR with ACEIs treatment, although without a significant reduction in the number of chest pain episodes.^133^ However, those findings should be interpreted cautiously because treatment strategy is closely related to hypertension, diabetes, heart failure, and other cardiovascular comorbidities, introducing potential confounding by indication.

#### Colchicine

Colchicine has widespread cellular effects, primarily through the inhibition of tubulin polymerization. This action modulates leukocyte responsiveness by decreasing IL-1β production through inactivation of the NLRP3 inflammasome pathway.^134^ Moreover, colchicine inhibits neutrophil extracellular trap formation and reduces myocardial inflammation.^135^ This capacity to modulate inflammatory responses may enhance myocardial recovery and prevent further injury due to MVO during acute ischaemic episodes.

The Colchicine Cardiovascular Outcomes Trial (COLCOT) showed that colchicine 0.5 mg per day reduces the risk of ischaemic cardiovascular events in patients with recent MI.^136^ Similarly, the Low-dose Colchicine 2 (LoDoCo2) trial demonstrated that low-dose colchicine (0.5 mg/day) decreases cardiovascular events in patients with chronic coronary disease.^137^ A pilot randomized clinical trial suggested that colchicine may improve coronary microvascular function, as evidenced by a smaller procedure-related troponin increase and a lower neutrophil count after percutaneous coronary intervention (PCI) in patients with stable angina or non-ST-elevation myocardial infarction (NSTEMI). However, colchicine treatment before PCI did not significantly affect CFR or resistive reserve ratio (RRR).^138^ Conversely, the CLEAR SYNERGY trial found no significant differences in cardiovascular death, MI, stroke, or the need for further revascularization in patients treated with colchicine and those receiving placebo. Although inflammation markers were reduced, colchicine did not provide clinical benefit, and side effects like diarrhoea were more frequent. These results do not support the routine use of colchicine after MI.^139^ Despite evidence supporting IL-1β pathway modulation in reducing systemic inflammation, no trials to date have specifically evaluated its impact on coronary microvascular physiology, leaving its relevance to CMD speculative. Further studies are needed to clarify the relationship between the anti-inflammatory effects of colchicine and CMD.

Targeted cytokine inhibition has also been explored in atherosclerotic cardiovascular disease. IL-1β pathway modulation has demonstrated outcome benefits in selected high-risk populations, supporting the inflammatory hypothesis of atherosclerosis. However, no clinical trials have specifically evaluated the impact of IL-1β inhibition on invasive or imaging-defined coronary microvascular dysfunction endpoints. As such, its relevance to CMD remains biologically plausible but clinically unproven.

### Emerging therapeutic strategies

Some new therapies have preliminarily demonstrated multiple anti-inflammatories and pleiotropic cardiovascular benefit in broad populations such as diabetes and heart failure. However, direct evidence targeting CMD endpoints remains limited. Importantly, the biological rationale for anti-inflammatory intervention may differ across CMD phenotypes, including structural remodelling, functional vasomotor dysfunction, and post-MI MVO. Therefore, when interpreting therapeutic evidence in CMD, it is important to distinguish between biomarker changes, endothelial function, coronary physiological endpoints such as CFR or IMR, symptoms including angina and quality of life, heart failure outcomes, and survival. Many therapies discussed below have demonstrated cardiovascular benefit in broader populations, but few have been tested in dedicated CMD trials using invasive or imaging-defined coronary microvascular endpoints. Also, treatment allocation is frequently influenced by underlying cardiovascular risk, comorbidities, disease severity, and concomitant therapies, while systemic inflammation may represent either a contributor to or a consequence of CMD. Therefore, observed associations between therapeutic exposure, inflammatory biomarkers, and coronary microvascular function should not necessarily be interpreted as causal. Therapeutic implications are presented as hypothesis-generating rather than established CMD treatment recommendations.

#### Glucagon-like peptide-1 receptor agonists

Glucagon-like peptide-1 receptor agonists (GLP-1 RAs) have been investigated for potential anti-inflammatory and endothelial-modulating effects in metabolic and cardiovascular disease as promising agents with anti-inflammatory properties, contributing to their beneficial effects in metabolic and cardiovascular diseases, including CMD.^140^ GLP-1 RAs anti-inflammatory actions may include the modulation of the NF-κB pathway and inhibition of ROS formation.^141^ Some studies have shown that dulaglutide reduces cardiovascular events in patients affected by type 2 diabetes mellitus (T2DM) with or without established cardiovascular disease,^142^ while liraglutide demonstrated similar benefits in subjects with a history of MI or stroke.^143^ Based on the bulk of clinical evidence, the American Diabetes Association and the European Association for the Study of Diabetes recommend GLP-1RAs as the first-line treatment for patients with T2DM and established cardiovascular disease.^144^ In a study involving 85 patients with STEMI, those who received liraglutide 30 min before PCI and continued treatment for 7 days post-procedure exhibited a lower incidence of no-flow, as assessed by transthoracic Doppler echocardiography (TTDE). Although the difference was not statistically significant, notable reductions in inflammation markers such as high-sensitivity C-reactive protein (hs-CRP) and IL-6 were observed. Additionally, improvements in endothelial function, reflected by increased eNOS activity and elevated serum NO levels, highlighted differences between the GLP-1 RAs group and the control group.^145^ Furthermore, one study involving 31 newly diagnosed T2DM patients suggested that coronary flow velocity reserve (CFVR), assessed by TTDE, improved after 12 weeks of exenatide treatment, accompanied by reductions in ICAM and VCAM.^146^ This study provided evidence that exenatide was associated with improvement in endothelial function by decreasing ICAM-1 and ROS levels and up-regulating NO and eNOS production. However, the Functional Impact of GLP-1 RAs for Heart Failure Treatment (FIGHT) trial showed no difference in mortality or heart failure-related hospitalization rates between liraglutide and placebo treatment. Furthermore, in a double-blind, randomized, placebo-controlled study, MBF and MFR have no significant differences after 24 weeks of liraglutide treatment in chronic heart failure patients without diabetes.^147^ These findings suggest that the cardiovascular benefits of GLP-1 RAs may be attenuated in patients with advanced heart failure, rather than indicating an adverse effect. Whether GLP-1 RAs can treat CMD in patients with or without T2DM by modulating inflammation is still unclear. Although GLP-1 RAs may influence pathways linked to anti-inflammatory and endothelial-protective effects and reduce major adverse cardiovascular events in high-risk populations, dedicated CMD-targeted trials evaluating CFR or microvascular resistance are scarce. Thus, their application in CMD remains hypothesis-generating rather than guideline-directed. Further research is warranted to elucidate the specific effects of GLP-1 RAs on coronary microvascular function and inflammation in this clinical setting.

#### Sodium-glucose cotransporter-2 inhibitors

Sodium-glucose cotransporter-2 inhibitors (SGLT2i) reduce glucose reabsorption in the proximal renal tubule.^148^ Initially developed for the management of T2DM, SGLT2i have demonstrated cardiovascular and renal benefits in major outcome trials.^149,150^ Dapagliflozin has been shown to reduce the risk of heart failure-related hospitalizations in patients with T2DM and either established cardiovascular disease or multiple cardiovascular risk factors. Notably, this suggests benefits beyond its glucose-lowering effects, with a similar protective action in patients regardless of diabetes status.^151^ Experimental and preclinical studies suggest that SGLT2i may modulate pathways associated with vascular inflammation, oxidative stress, endothelial dysfunction, and cytokine activation. These effects have been linked to preservation of endothelial integrity and improved microvascular function in experimental models.^152^ They also preserve microvascular endothelial integrity and attenuate endoplasmic reticulum stress caused by glycocalyx disruption.^153^ In an animal model, empagliflozin was shown to protect against diabetic CMD by improving CFR, enhancing microvascular density, and reducing cardiac ﬁbrosis.^74^ In human internal thoracic artery and left ventricle specimens, low-grade inflammation increases SGLT2 mRNA and protein expression, leading to eNOS-NO/ROS imbalance, which can be counteracted by SGLT2i.^154^ Additionally, SGLT2i may modulate pathways associated with enhanced NO bioavailability and reduced oxidative stress, which corresponds to lower expression levels of ICAM-1 and VCAM-1.^155,156^ The observed endothelial and anti-inflammatory effects of SGLT2i provide mechanistic rationale for potential relevance to CMD; however, direct CMD-specific clinical evidence remains limited. While SGLT2i have consistently improved cardiovascular and heart failure outcomes, robust randomized trials specifically assessing invasive or imaging-defined CMD endpoints remain limited. Current support for their role in CMD is therefore primarily indirect and mechanistic, with emerging but not definitive CMD-focused data. Continued research will likely further explore these mechanisms, elucidating the comprehensive cardiovascular benefits of SGLT2 inhibition.

#### Tumour necrosis factor-α inhibitors

TNF-α plays a significant role in the development of endothelial dysfunction by promoting vascular inflammation, oxidative stress, and impaired vasodilation.^20^ TNF-α inhibitors (e.g. Etanercept, Infliximab) block TNF-α activity, thereby reducing inflammation. A study demonstrated that TNF-α inhibitors suggest potential improvement in endothelial or microvascular function in patients with established psoriasis refractory to conventional long-term therapy.^157^ These findings suggest that therapies targeting systemic inflammation may have a beneficial impact on coronary microvascular function. Small mechanistic studies suggest potential improvement in endothelial function; however, high-quality randomized evidence assessing microvascular haemodynamics in CMD populations is lacking.

Preliminary clinical studies have explored various pharmacological therapies that aim to improve CMD through anti-inflammatory mechanisms, as outlined in *Table 3*.

**Table 3:** Studies evaluating therapies with potential relevance to inflammatory pathways and coronary microvascular function

Pharmacological intervention	References	Study subjects	Study design	Methods (diagnostic modality)	Duration of the treatment	Findings
Statins	Jingwen Yong *et al.* 2020^129^	252 patients	10 studies meta-analysis			Patients received statins treatment have a significant improvement in CFR
ACE inhibitors	Jingwen Yong *et al.* 2020^129^		10 studies meta-analysis			CFR improved in patients with angina
	Mohamed R Abouzid *et al.* 2024^133^	158 patients	5 studies meta-analysis			CFR improved in CMD patients with angina, but not in the number of chest pain episodes
ARBs	Jingwen Yong *et al.* 2020^129^		9 studies meta-analysis			CFR improved in patients with angina
Colchicine	Justin Cole *et al.* 2022^138^	50 patients	Randomized, placebo-controlled pilot study	Pressure-flow wire FFR, IMR, CFR, both pre- and post-PCI	6–24 h before PCI	RRR and CFR improved, but not statistically significant in modifying PCI-related CMD
GLP-receptor agonists	Chen, W.R 2015^145^	85 STEMI patients	Randomized, placebo-controlled	TTDE	7 days liraglutide	Lower rate of non-reflow
	Wei, R. *et al.* 2016^146^	31 T2DM	Placebo-controlled	TTDE	12 weeks exenatide	Improved CFVR; ICAM and VCAM decreased
	Nielsen, R. 2019^147^	36 HFrEF without Diabetes	A double-blind, randomized, placebo-controlled	^15^O-H_2_O PET/CT	24 weeks	No improvement in MBF or MFR
SGLT2i	Katrine M. Lauritsen *et al.* 2021^158^	13 T2DM	Randomized double-blind, placebo-controlled crossover study	^15^O-H_2_O PET/CT	4 weeks	Rest MBF decreased by 13%, but did not significantly change stress MBF or MFR
	Hannah Elena Suhrs *et al.* 2022^159^	19 T2DM	Randomized, placebo-controlled cross-over study	TTDE	12 weeks	No improvement in CFVR
	Mikkel Jürgens *et al.* 2021^160^	90 T2DM with high cardiovascular risk	Prospective randomized, the SIMPLE Trial	Rubidium-82 PET/CT	13 weeks	No effect on MFR
	Umberto Capece and Chiara Pavanello *et al.* 2024^161^	16 T2DM with CAD	Prospective randomized, the DAPAHEART-Trial	13N-ammonia PET/CT	4 weeks	Dapagliflozin significantly improved MFR
	Ali Mroueh *et al.* 2024^154^	70 CAD with/without T2DM	Cross-sectional study	CT scan and echocardiography		Low-grade inflammation was associated with altered SGLT2 expression and function and with TNF-α-related changes in SGLT2 activity
TNF-α inhibitors	Stefano Piaserico *et al.* 2016^157^	37 patients with severe psoriasis	Prospective self-controlled	TTDE	6.3 months (range 5.5–7.1 months)	CFVR improved

ACE, angiotensin-converting enzyme; ARBs, angiotensin II receptor blockers; CAD, coronary artery disease; CFR, coronary flow reserve; CFVR, coronary flow velocity reserve; CMD, coronary microvascular dysfunction; CT, computed tomography; DAPAHEART-Trial, Dapagliflozin Improves Myocardial Flow Reserve in Patients with Type 2 Diabetes; FFR, fractional flow reserve; HFrEF, heart failure with reduced ejection fraction; ICAM, intercellular adhesion molecule; IMR, index of microcirculatory resistance; MBF, myocardial blood flow; MFR, myocardial flow reserve; PET/CT, positron emission tomography/computed tomography; RRR, resistive reserve ratio; SGLT2, sodium-glucose cotransporter-2; SGLT2i, sodium-glucose cotransporter-2 inhibitor; SIMPLE Trial, Shockless IMPLant Evaluation Trial; STEMI, ST-segment elevation myocardial infarction; T2DM, type 2 diabetes mellitus; TNF-α, tumour necrosis factor-α; TTDE, transthoracic Doppler echocardiography; VCAM, vascular cell adhesion molecule.

The potential molecular pathways and therapeutic strategies relevant to inflammation in CMD have been summarized in *Figure 3*. Collectively, current therapeutic evidence highlights a gap between mechanistic plausibility and CMD-specific clinical validation. Future studies should prioritize specific trial designs incorporating invasive and non-invasive microvascular endpoints, with careful differentiation between structural and functional CMD phenotypes. Until such data are available, anti-inflammatory strategies in CMD should be considered biologically rational but incompletely validated.

**Figure 3:**
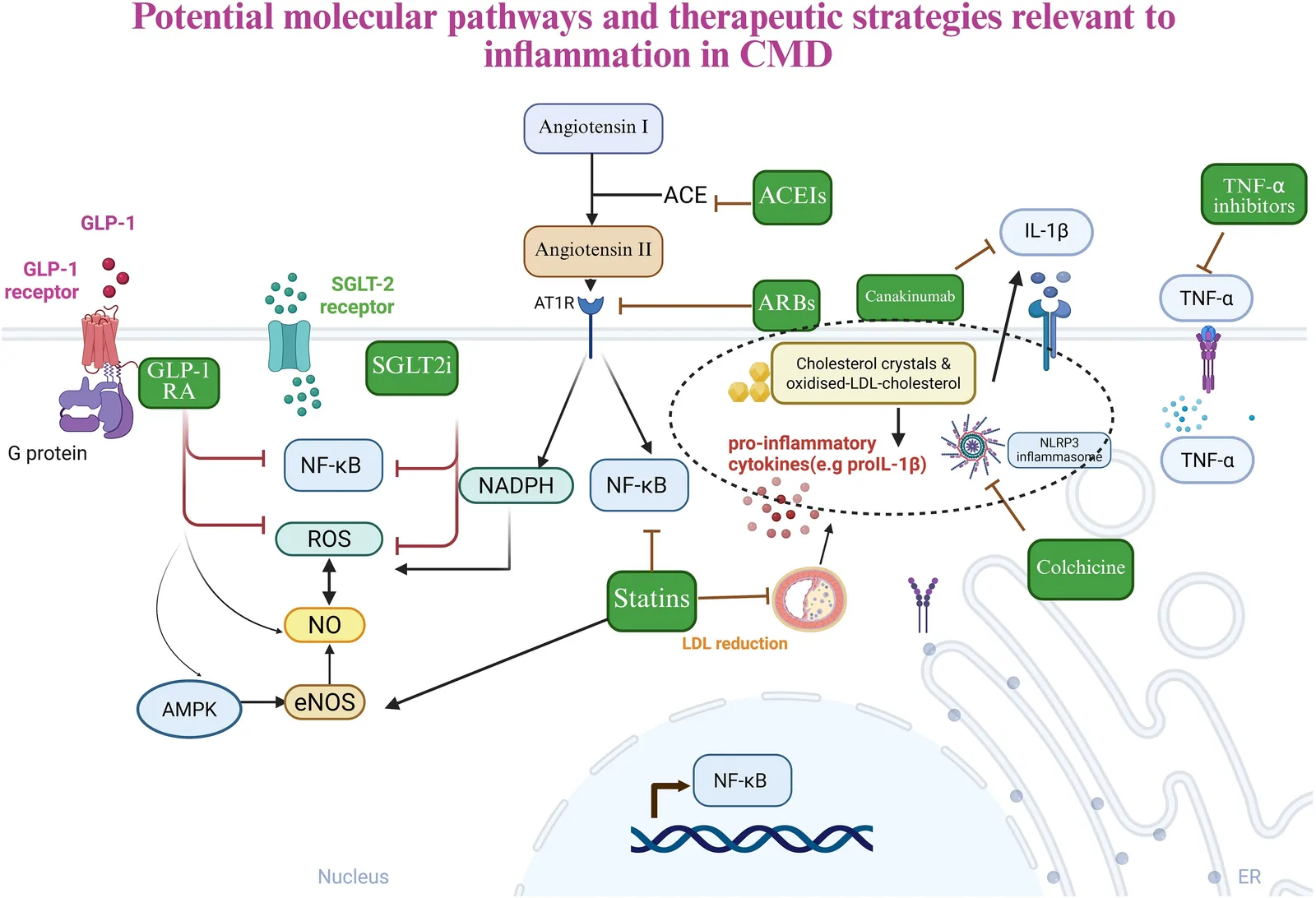
Potential molecular pathways and therapeutic strategies relevant to inflammation in CMD. ACE, angiotensin-converting enzyme; ACEIs, angiotensin-converting enzyme inhibitors; AMPK, AMP-activated protein kinase; ARBs, angiotensin II receptor blockers; AT1R, angiotensin II type 1 receptor; eNOS, endothelial nitric oxide synthase; ER, endoplasmic reticulum; GLP-1, glucagon-like peptide-1; GLP-1 RA, glucagon-like peptide-1 receptor agonist; GLP-1 receptor, glucagon-like peptide-1 receptor; IL-1β, interleukin 1 beta; NADPH, nicotinamide adenine dinucleotide phosphate; NF-κB, nuclear factor kappa B; NLRP3, NOD-, LRR-, and pyrin domain-containing protein 3; NO, nitric oxide; ROS, reactive oxygen species; SGLT2 receptor, sodium-glucose cotransporter 2 receptor; SGLT2i, sodium-glucose cotransporter 2 inhibitors; TNF-α, tumour necrosis factor-α.

## Non-pharmacological approaches

Lifestyle modification could be framed as improving cardiometabolic risk and endothelial health, rather than directly treating CMD, because there is limited evidence of CMD-specific CFR/IMR studies. Dietary modifications and regular exercise are essential for improving overall cardiovascular health. These interventions improve cardiometabolic risk factors and endothelial function and may benefit coronary microvascular function.^162^ The Mediterranean diet—rich in healthy fats, fruits, vegetables, and whole grains—is recognized for its anti-inflammatory properties. Dietary patterns reducing refined sugars and saturated fats have been associated with improved endothelial function and reduced vascular inflammation.^163^ Furthermore, regular moderate-to-high-intensity aerobic exercise may further enhance these effects through loss of body fat and cardiorespiratory fitness.^164^ Collectively, these lifestyle changes significantly improve insulin sensitivity, ultimately alleviating symptoms associated with myocardial ischaemia.

## Future research directions

CMD is gaining increasing academic attention as a key contributor to IHD, particularly in patients with INOCA and HFpEF. Evidence from both basic and clinical research supports a crucial role of inflammation in CMD, although many aspects—from pathophysiology to treatment—remain only partially understood. Future studies should clarify how chronic inflammation and immune cell interactions vary according to sex and age and aim to map inflammatory cell subsets within the coronary microcirculation. Furthermore, further research is needed to identify circulating biomarkers and imaging modalities that reflect coronary microvascular inflammatory activity and assess CMD severity or treatment response. Translational investigations should further evaluate anti-inflammatory and metabolic therapies, including SGLT2i, GLP-1 RAs, and IL-1 or IL-6 pathway modulators, in improving CMD outcomes. Collectively, these approaches could help clinicians identify patients with CMD at an early stage and promote more precise, targeted treatments to improve their prognosis.

## Conclusion

Chronic inflammation appears to contribute variably to the pathophysiology of CMD, functioning in different clinical contexts as a potential driver of structural injury, an amplifier of vasomotor dysregulation, or a downstream consequence of systemic cardiometabolic stress. Recognition of this biological and phenotypic heterogeneity is essential for refining diagnostic strategies and for interpreting therapeutic interventions targeting inflammatory pathways. Although mechanistic and translational studies support a biological link between inflammation and CMD, robust clinical evidence specifically demonstrating improvement in coronary microvascular haemodynamics remains limited. Furthermore, findings derived from post-MI microvascular obstruction, type 2 diabetes, HFpEF, autoimmune disease, or imaging-defined inflammatory cohorts may not be generalizable across all CMD phenotypes. Future research should prioritize phenotype-specific study designs integrating mechanistic biomarkers, invasive and non-invasive microvascular assessment, and longitudinal outcome evaluation to better identify patient subgroups most likely to benefit from targeted anti-inflammatory approaches.

## Data Availability

This article is a narrative review based on previously published studies. No new datasets were generated or analysed during the current study.
